# Barriers to physical activity and patient profiling in the lifelong maintenance phase of coronary artery disease: a territorial mixed-methods pilot study

**DOI:** 10.3389/fresc.2025.1659925

**Published:** 2025-10-01

**Authors:** Morgane Molina, Fabienne Durand, Henri Meric

**Affiliations:** ESPACE DEV, Universite de Perpignan Via Domitia, Perpignan, France

**Keywords:** cardiac rehabilitation, physical activity, lifelong maintenance phase, coronary artery disease, machine learning, delphi method

## Abstract

**Introduction:**

Despite proven benefits, adherence to physical activity (PA) during the lifelong maintenance phase of cardiac rehabilitation (CR) remains suboptimal. Understanding territorial-specific barriers is essential for developing targeted interventions. This pilot study aimed to (1) identify principal barriers to PA among coronary artery disease (CAD) patients in lifelong maintenance Phase CR in a specific territory of southern France, and (2) characterize distinct patient profiles using unsupervised machine learning.

**Methods:**

Socio-demographic data, completion of a full Phase II CR and behavioral characteristics related to PA during the lifelong maintenance Phase were collected with a LimeSurvey questionnaire. A modified Delphi method was employed with CAD patients (*n* = 26, subsequently, *n* = 13 in round 2) who had completed a supervised Phase II CR. Barriers were categorized and ranked using Likert scales. K-means clustering analysis was then applied to identify homogeneous patient subgroups based on barrier patterns.

**Results:**

Nine barrier categories emerged, with environment (8.3 ± 1.0), motivation (7.7 ± 1.4), and exercise tolerance (6.3 ± 1.2) ranking highest. Kendall's W = 0.64 (*p* < 0.001) indicated a significant consensus. Three cluster were identified: Cluster 1 (38.46%) characterized by physical deconditioning; Cluster 2 (23.07%) by environmental and motivational constraints; Cluster 3 (38.46%) by organizational limitations. Significant between-cluster differences were observed for: environmental barriers (H = 7.82, *p* = 0.02), motivation (H = 8.14, *p* = 0.017), and professional obligations (H = 6.93, *p* = 0.031).

**Conclusion:**

This mixed-methods approach revealed complex, interrelated barriers to PA maintenance. The identification of distinct CAD patient profiles suggests that personalized intervention strategies, rather than one-size-fits-all approaches, may enhance long-term adherence to PA in lifelong maintenance Phase CR.

## Introduction

Advances in cardiovascular medicine have markedly improved survival rates among individuals with coronary artery disease (CAD) through early detection, evidence-based treatments, and management via cardiac rehabilitation (CR) (1). CR programs are a cornerstone of these strategies, aiming to reduce cardiovascular morbidity and mortality through the management of modifiable risk factors such as sedentary behavior, smoking, dyslipidemia, and hypertension (2). CR is typically structured in three to four progressive phases, depending on national practices: Phase I begins during hospitalization and focuses on restoring basic functional capacity; Phase II takes place shortly after discharge and involves structured, supervised exercise and risk factor education. Phase III is a maintenance phase widely recognized as a key element in the continuity of care for patients with cardiovascular diseases. In some countries, phase IV is distinguished as a continuation of Phase III with less supervision, often delivered in community centers or independently (3, 4). Although initially designed for patients with CAD, the lifelong-maintenance phase (III/IV) CR is increasingly extended to individuals with other chronic conditions, such as heart failure, peripheral artery disease, or diabetes (5). Generally delivered over an average of nine weeks with two sessions per week, lifelong maintenance Phase targets sustained behavioral change through ongoing physical activity (PA) and lifestyle adherence in outpatient or community-based settings. Despite the wide availability of its programs in 89% of countries, these are often limited to a small number of groups with the notable exception of Germany, which has a particularly well-developed system (3). However, as Lion et al. points out, significant variability exists in how these programs are implemented across different regions, highlighting inconsistencies in referral processes, medical oversight, and program structure (6). According to Pesah et al, regular PA remains a cornerstone of cardiac rehabilitation, playing a key role in enhancing cardiorespiratory fitness, psychological well-being, and long-term clinical outcomes (5).

However, despite the well-documented benefits of regular PA in reducing the risk of recurrent cardiac events, adherence to exercise tends to decline sharply after Phase II (7). Numerous studies have identified a range of barriers to sustained engagement, which can be grouped into a multidimensional framework integrating various factors (8–10). These include: (a) Intrapersonal: physical limitations, low motivation, misconceptions about lifelong maintenance Phase, low self-efficacy, and comorbidities; (b) Interpersonal: lack of social support, family obligations, and caregiving responsibilities; c) Environmental: poor access to exercise facilities, transportation issues, distance, and weather; (d) Organizational/systemic: lack of referral from healthcare professionals, inflexible schedules, limited availability of programs, and insufficient long-term follow-up; e) Socioeconomic: cost-related constraints, professional reintegration, and unequal access based on geographical location or socioeconomic status. In a recent study conducted in Portugal, Santos Fonseca et al. describe the typical profile of patients enrolled in lifelong Phase III cardiac rehabilitation programs as predominantly male, married, and either employed full-time or retired (11). Most had transitioned from a Phase II program, were highly educated, and lived in close proximity to the CR facilities, which likely contributed to their continued participation and high levels of adherence.

In France, CAD patients benefit from comprehensive coverage under the Long-Term Illness (Affection de Longue Durée, ALD) scheme. Although the initial phases of CR are covered by national health insurance, there is currently no fully reimbursed, structured program for lifelong maintenance of CR at the national level. Furthermore, the recent introduction of PA prescriptions within the healthcare system has improved access to structured, professionally supervised exercise programs, with partial reimbursement available depending on the type of healthcare professional involved. Nonetheless, adherence to the lifelong-maintenance Phase remains suboptimal, reflecting broader European patterns of underutilization and inequality in program availability (4, 10)

While the south of France is well represented by its Mediterranean climate and large cities with health and socio-economic facilities, the Eastern Pyrenees department stands apart. Situated within a socio-economically disadvantaged context due to the lack of major companies and industries, this department nevertheless possesses healthcare facilities that locally provide management of Phases I and II CR. Both social and meteorological conditions may either facilitate or impede the practice of outdoor PA. In this regard, the Eastern Pyrenees is characterized by a generally warm yet windy climate. Understanding context-specific barriers and identifying patient subgroups most at risk of dropout is essential for tailoring sustainable interventions. While some research has incorporated patient perspectives (12, 13), participatory approaches remain underused in the context of lifelong CR for CAD (14, 15). Indeed, a growing body of literature highlights the heterogeneity of patients engaged in lifelong CR, including differences in age, sex, comorbidities, occupational status, and psychosocial characteristics (11).

Accordingly, this study aimed to achieve two primary objectives:

- First, to identify and prioritize the main barriers to physical activity engagement during the lifelong maintenance phase among patients with CAD living in the Eastern Pyrenees. In this regard, exploring patients lived experiences constitutes both a methodologically rigorous and ethically justified approach, as it provides critical insights necessary for the development of patient-centered and contextually relevant interventions.
- Secondly, to delineate distinct patient profiles to better understand patterns of adherence using unsupervised machine learning techniques, and guide the development of targeted, context-specific interventions.

## Materials and methods

### Study design

This pilot study was conducted to assess the feasibility of a larger-scale investigation with broader relevance beyond the Eastern Pyrenees region. It employed a mixed-methods approach, combining a modified Delphi process (qualitative item generation in round 1; quantitative ranking in round 2) with exploratory clustering analysis to identify and characterize patterns of barriers among patients with CAD in the lifelong maintenance phase. In addition to the stated scientific objectives, this study format allowed for the evaluation of participant recruitment methods, the verification of data collection procedures, and the assessment of protocol adherence by both clinicians and patients particularly by identifying potential challenges in implementing the protocol in a real-world setting (16). The study protocol received approval from the Ethics Committee of Sport Sciences Research (CER-STAPS 00012476-2024-04-03-297). Data collection was conducted between June and November 2024.

For this pilot study, recruitment of 100 participants was conducted through targeted email distribution based on contact databases from partner rehabilitation clinics and patient advocacy networks within the Eastern Pyrenees. Inclusion criteria comprised: French-speaking adults (≥18 years), confirmed CAD diagnosis by a medical practitioner and completion of structured Phase II CR. Exclusion criteria, assessed through initial questionnaire responses, included absence of medically confirmed CAD diagnosis or lack of participation in formal Phase II CR programs.

### Initial questionnaire

All participants provided electronic informed consent integrated within the initial questionnaire. This questionnaire collected socio-demographic data (age, sex, marital status, number of children, employment status, and environment of residence (urban, semi-urban, rural), clinical information (confirmation of CAD diagnosis by a physician and completion of a full Phase II CR program), prior participation in supervised lifelong maintenance Phase CR and behavioral characteristics related to physical activity: perceived health benefits of PA, current PA practice, frequency of practice, duration of sessions, location of practice, whether PA was performed alone, with relatives, or under professional supervision (adapted physical activity APA professional or not).

### Modified delphi method

Well-suited to identifying barriers to sustained physical activity in CAD patients, who are regarded as experts of their own experience (17), the Delphi process typically consists of three iterative rounds in which participants rate items, receive anonymized group feedback, and then re-evaluate their responses until consensus is reached (18). In some cases, however, the process can be stopped after two rounds if consensus has been sufficiently achieved or if further rounds are unlikely to modify the results (19). Whereas traditional Delphi studies typically rely on a ≥ 75% agreement threshold, this study employed a modified Delphi approach using Kendall's W coefficient (≥0.50) to assess consensus. This choice aligns with contemporary recommendations for exploratory health research, where moderate levels of agreement are considered acceptable for the initial identification of barriers (20, 21). In our study, a real-time Delphi survey was administered using LimeSurvey software (22), and organized as follows: Round 1 incorporated the initial electronic questionnaire and email collection for subsequent rounds and the central open-ended question: “In your opinion, what are the main factors that hinder your regular physical activity and should be addressed to help improve your engagement in physical activity?” Responses underwent thematic analysis with categorization based on content similarity and frequency counts. Categories representing the most prevalent themes were retained for subsequent rounds to maintain methodological feasibility. Round 2: Participants who completed round 1 in their entirety were invited to participate. Those providing incomplete responses or withdrawing consent were excluded. The synthesized barrier categories from round 1 were presented with descriptive definitions, without revealing frequency data to avoid anchoring bias. Participants indicated their agreement level using a 10-point Likert scale (1 = least important barrier; 10 = most important barrier) for the statement: “These factors prevent you from maintaining regular physical activity after rehabilitation. Please indicate your level of agreement with each statement.” An optional free-text field allowed additional commentary. The implementation of round 3 was contingent upon the absence of consensus in round 2. It followed the same procedures, with the exception that the lowest-ranked categories were eliminated. Participants received aggregated data from round 2, including mean rankings, to guide their final prioritization of barriers in order of restrictiveness. The modified Delphi method relies on full participation from experts in each round. Including partial responses would compromise the methodological rigor of the study (23).

### Unsupervised machine learning method (K-means)

To identify underlying patterns within the data, K-means clustering was used. Optimized for identifying spherical data patterns (24), the clustering methodologies enable cross-referential data analysis. To determine the optimal number of clusters (k), we employed both the elbow method and silhouette analysis. However, given that the silhouette score provides a more objective means of determining the optimal number of clusters particularly when “the elbow curve is not sufficient to find the right ‘K'" (25), we prioritized the silhouette coefficient for final determination. The silhouette score measures how similar an object is to its own cluster compared to other clusters, with values ranging from −1 to 1, where higher values indicate better-defined clusters. The choice of K-means over alternative clustering methods was justified by several factors. First, K-means demonstrates computational efficiency for moderate-sized datasets. Second, recent cardiovascular research has shown that K-means clustering achieved the highest predictive accuracy (0.8598) compared to other machine learning methods (26).

K-means clustering was applied exclusively to the numerical Likert scale ratings (1–10) from Round 2, treating these as continuous variables following standard practice in behavioral research (27). No categorical variables were included in the clustering algorithm. Demographic categorical variables (gender, age groups, professional categories) were used only for *post-hoc* cluster characterization through cross-tabulation, not in the clustering process itself.

### Statistical analysis

Descriptive statistics were used to describe sociodemographic data and calculate the degree of consensus for each item of modified Delphi process (28, 29). At the end of this last round, a Kendall's coefficient was calculated to establish the level of consensus among the 13 expert patients. Spearman rank correlation coefficients were calculated between all nine barrier importance ratings from Round 2 to explore interdependencies between perceived obstacles. This non-parametric approach was selected due to the ordinal nature of Likert data and non-normal distributions (assessed by Shapiro–Wilk test). Correlation strength was interpreted as: r > 0.6 = strong, 0.4–0.6 = moderate, <0.4 = weak.

Between-group comparisons of continuous variables employed the Kruskal–Wallis test, appropriate given non-Gaussian distributions and the exploratory nature of unsupervised classification.

All analyses were performed using RStudio (version 4.4.3). Significance was set at *p* < 0.05.

## Results

**Figure 1:**
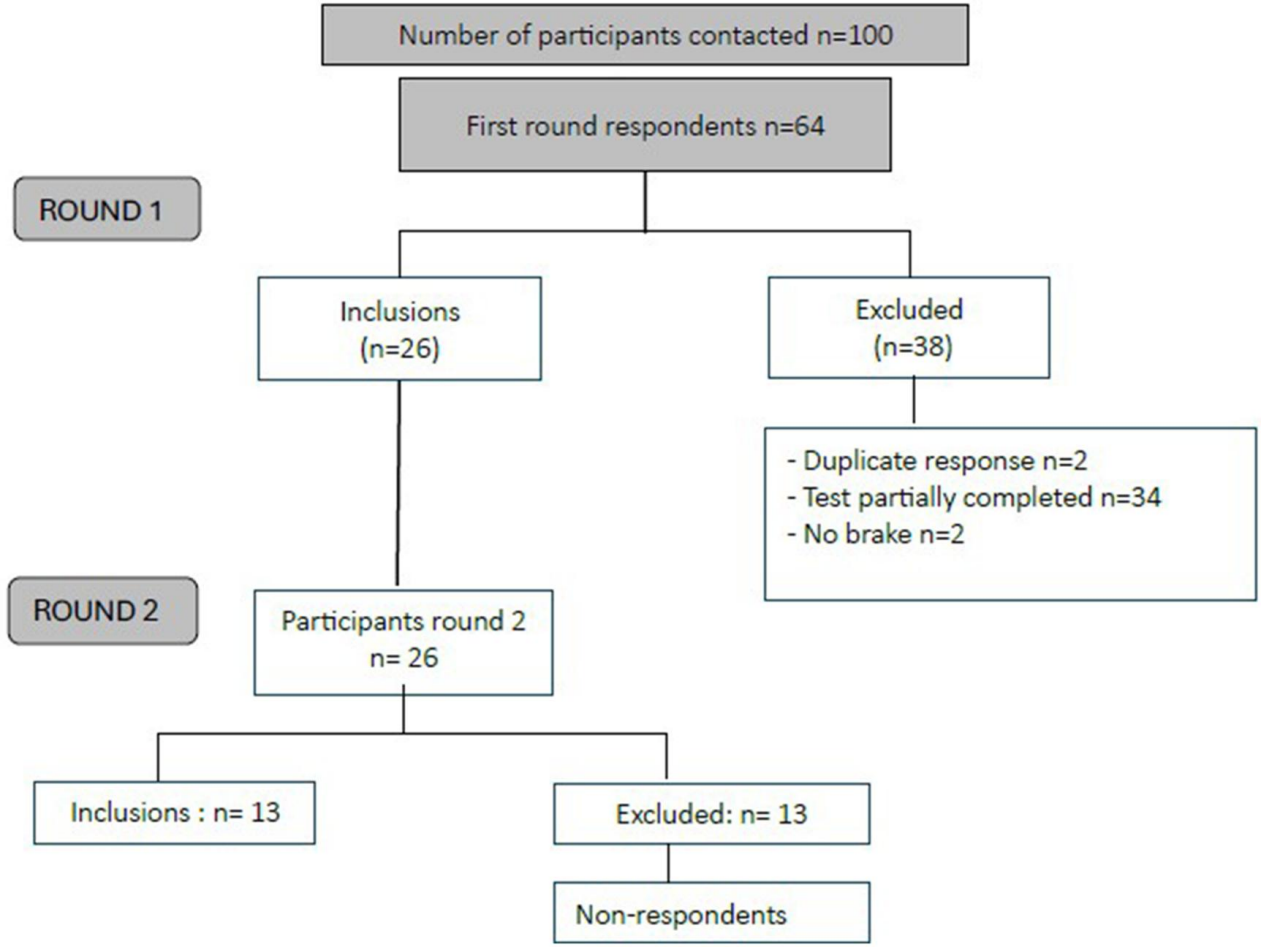
Inclusion and exclusion flowchart.

Figure 1 presents the participant flow through both Delphi rounds. Of the 100 questionnaires distributed, 36 were excluded due to the absence of a confirmed CAD diagnosis, no prior participation in Phase II cardiac rehabilitation, or a complete lack of response. Among the 64 responses collected, 2 were identified as duplicates, 2 indicated the absence of barriers to PA, and 34 were incomplete, yielding a final sample of 26 participants for the subsequent round of the Delphi process.

Table 1 summarizes participant socio-demographics. Briefly, the cohort was predominantly male (92.3%), aged over 50 years (mean 63.7 ± 8.7 years), and retired. Most participants possessed a high level of education, with a substantial proportion employed in intellectual or intermediate-level professions. All participants had completed a structured Phase II cardiac rehabilitation program, with the vast majority having done so within the preceding year. Modalities and level of PA practice was reported in Table 2.

**Table 1:** Socio-demographic data of the whole population (*n* = 26, round 1).

Variable	*n*	%
Gender		
Men	24	92.31
Women	2	7.69
Age (years)		
30–40	0	0
40–50	1	3.85
50–60	9	34.62
>60	16	61.54
Completion of supervised Phase II CR (months)		
Less than 6	13	50
Between 6 and 12	11	42.31
Between 12 and 24	1	3.84
More than 24	1	3.84
Occupational status		
Employed	8	30.76
On sick leave	2	7.69
Retired	16	61.53
Socio-professional category		
Farmers	1	3.85
Craftsmen and shopkeepers, company directors	1	3.85
Executives and intellectual professions	12	46.15
Intermediate professions (health, police, teachers..)	4	15.38
Employees	5	19.23
Workers	3	11.53
Place of residence (multiple choice)		
Urban	11	40.74
Rural	7	25.92
Coastal	5	18.51
Plaine	2	7.4
Foothills/ mountainous hinterland	2	7.4
Highlands/mountain	0	0

**Table 2:** Modalities and level of PA practice of the whole population (*n* = 26, round 1).

Variable	*n*	%
Perceived benefits of PA		
Yes	26	100
Regular PA practice		
Yes	25	96.15
No	1	3.84
Frequency of PA (per week)		
0	0	0
1–2	6	23.07
2–4	14	53.84
>4	6	23.08
Average session duration		
30 min	10	38.46
45 min	3	11.53
1 h	6	23.07
>1 h	7	26.92
Main location of PA practice (multiple choice)		
At home	11	27.5
In the neighborhood	10	25
In nature	9	22.5
Fitness center	6	15
Practice location in “Heart and Health” club	2	5
Practice in other association	1	2.5
Practice in a public park	1	2.5
Practice in non-specialized sports club	0	0
Accompanied during PA		
Alone	18	69.23
Most often accompanied	4	15.38
Sometimes accompanied	4	15.38
Accompanied by		
Relative (family,friend)	7	87.5
Adapted physical activity (APA) professional	0	0
Non-specialist coach	1	12.5

### First round of the delphi process and establishment of categories

**Figure 2:**
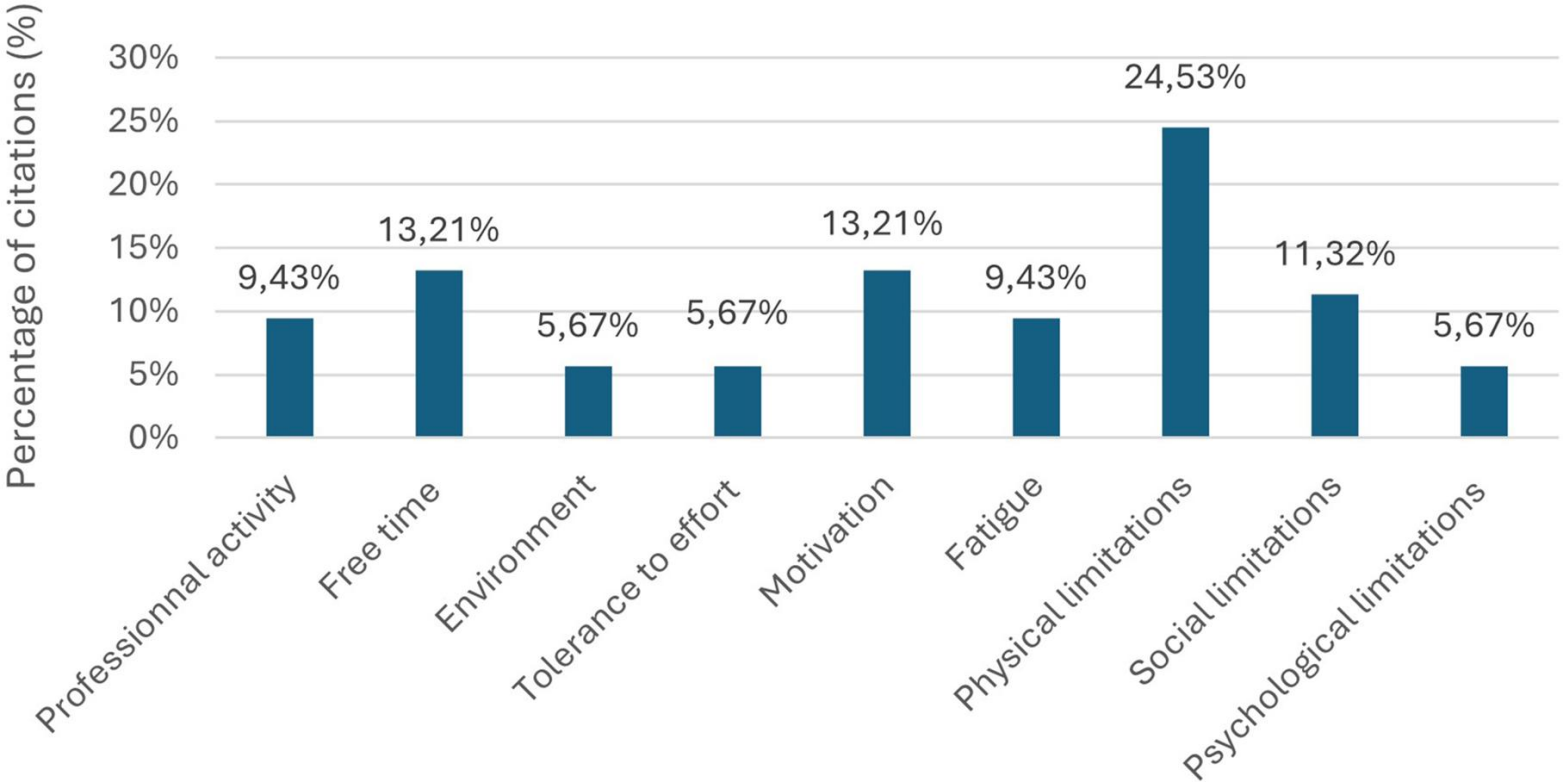
First round barrier categorization: percentage of citations by category from 53 identified barriers (*n* = 26). Response to: “In your opinion, what are the main factors that hinder your regular physical activity and should be addressed to help improve your engagement in physical activity?”.

The thematic analysis of the responses provided by the 26 participants in the first round of the Delphi process yielded 9 distinct barrier categories (Table 3). Physical limitations emerged as the most frequently cited impediment (24.53%), followed by environmental factors, time constraints, motivational challenges, exercise tolerance issues, occupational demands, psychological barriers, social limitations, and fatigue (Figure 2).

**Table 3:** Definitions of the 9 resulting categories after round 1.

Catégories	Definition
Professional activity	Constraints directly related to the participants’ professional activity (example: difficult trade such as masonry)
Free time	Time available to participants outside their work
Environment	Any element of the environment (access to infrastructure, access to a club, association, weather (hot, cold, wind, rain) that prevents the participant from performing regular physical activity.
Effort (exercise) tolerance	The ability of the participant to easily perform physical activity.
Motivation	The participant's desire to engage in regular physical activity for their health.
Fatigue	The participant's physical and/or mental fatigue preventing him from performing regular physical activity
Physical limitations	Physical problems that prevent the participant from performing regular physical activity but are not related to his or her coronary heart disease (example: Osteoarthritic pain)
Social limitations	Social and/or family life problems that prevent the participant from engaging in regular physical activity (example: caring for a sick relative)
Psychological limitations	Psychological problems that prevent the participant from performing regular physical activity (stress, attention disorder.)

### Second round of the delphi process, ranking and consensus

Of the 26 participants in the first round, only 13 proceeded to complete the second Round. No reasons for attrition were collected, as such inquiries are generally not conducted within the framework of a Delphi study. Tables 4, 5 respectively present socio-demographics and characteristics of PA practice of the 13 expert CAD patients.

**Table 4:** Socio-demographic data of the expert CAD patients (*n* = 13, round 2).

Variable	*n*	%
Gender		
Men	12	92.31
Women	1	7.69
Age (years)		
30–40	0	0.00
40–50	1	7.69
50–60	4	30.77
>60	8	61.54
Completion of supervised Phase II CR (months)		
Less than 6	6	46.2
Between 6 and 12	6	46.2
Between 12 and 24	0	0
More than 24	1	7.7
Occupational status		
Employed	8	61.54
On sick leave	2	15.38
Retired	3	23.08
Socio-professional category		
Farmers	1	7.69
Craftsmen and shopkeepers, company directors	1	7.69
Executives and intellectual professions	5	38.46
Intermediate professions (health, police, teachers..)	2	15.38
Employees	3	23.08
Workers	1	7.69
Place of residence *(multiple choice)*		
Urban	7	46.67
Rural	3	20
Coastal	1	6.66
Plaine	2	13.33
Foothills/ mountainous hinterland	2	13.33
Highlands/mountain	0	0

**Table 5:** Modalities and level of PA practice of the expert CAD patients (*n* = 13, round 2).

Variable	*n*	%
Perceived benefits of PA		
Yes	13	100.0
Regular PA practice		
Yes	12	92.3
No	1	7.69
Frequency of PA (per week)		
0	1	7.69
1–2	5	38.04
2–4	6	46.15
>4	1	7.69
Average session duration		
30 min	4	30.76
45 min	0	0
1 h	5	38.04
>1 h	4	30.76
Main location of PA practice *(multiple choice)*		
At home	6	35.29
In the neighborhood	5	29.41
In nature	5	29.41
Fitness center	1	5.88
Practice location in “Heart and Health” club	0	0
Practice in other association	0	0
Practice in a public park	0	0
Practice location in non-specialized sports club	0	0
Accompanied during PA *(multiple choice)*		
Alone	8	61.53
Most often accompanied	3	23.07
Sometimes accompanied	2	15.38
Accompanied by		
Relative (family,friend)	4	80
Adapted physical activity (APA) professional	0	0
Non-specialist coach	1	20

**Figure 3:**
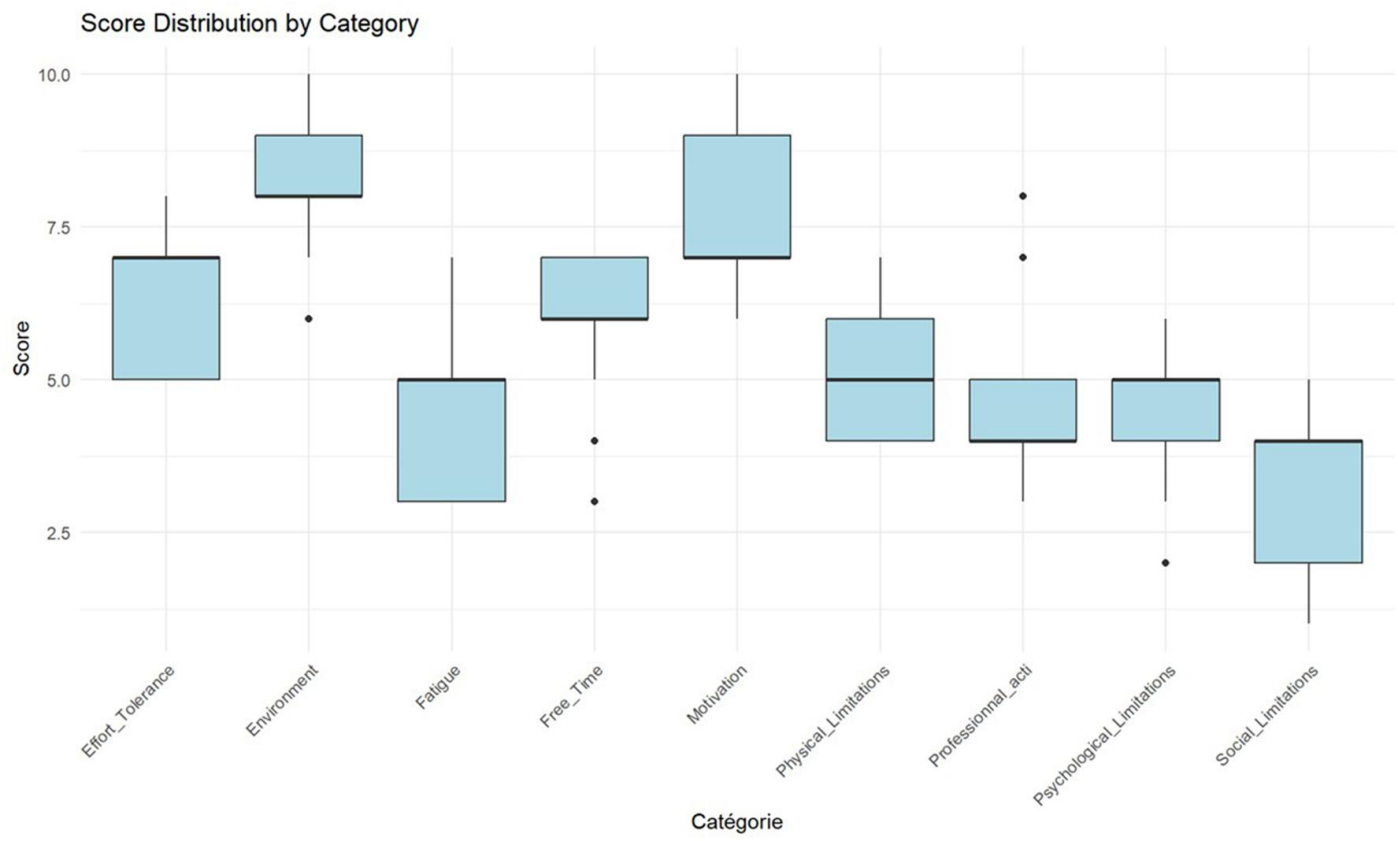
Second round ranking distribution (*n* = 13).

Figure 3 presents the rankings of barrier importance based on the 10-point Likert scale, revealing the following hierarchy of mean scores exceeding 5.0: environment (8.3 ± 1.0), motivation (7.7 ± 1.4), exercise tolerance (6.3 ± 1.2), free time (5.9 ± 1.2), and physical limitations (5.1 ± 0.9). Lower-priority categories included professional obligations (4.8 ± 1.3), psychological constraints (4.6 ± 1.5), fatigue (4.3 ± 1.2), and social limitations (3.3 ± 1.4). Spearman correlation analysis revealed statistically significant relationships between environmental barriers and exercise tolerance (r = −0.68, *p* = 0.01), as well as between environmental barriers and motivation (r = 0.61, *p* = 0.02). Environmental barriers demonstrated the highest inter-rater agreement. Kendall's coefficient of concordance yielded W = 0.64 (*χ*^2^ = 66.6, *p* < 0.001, *n* = 9, *N* = 13), indicating moderate yet statistically significant consensus.

### Clustering on round 2

K-means clustering applied exclusively to the numerical Likert scale ratings (1–10) from round 2, revealed 3 distinct patient profiles:

- Cluster 1: (*n* = 5; 38.46%): Characterized by severely compromised exercise tolerance (median = 9; IQR = 8–10), substantial physical limitations [8 (8–9)], and significant fatigue [7 (5–7)].
- Cluster 2: (*n* = 3; 23.07%): Distinguished by predominant environmental barriers [9 (8–10)], motivational deficits [9 (8–10)], and notable social constraints [7 (5–8)].
- Cluster 3: (*n* = 5; 38.46%): Defined by occupational impediments [7 (5–8)], time scarcity [7 (6–8)], and psychological challenges [6 (6–7)].

**Figure 4:**
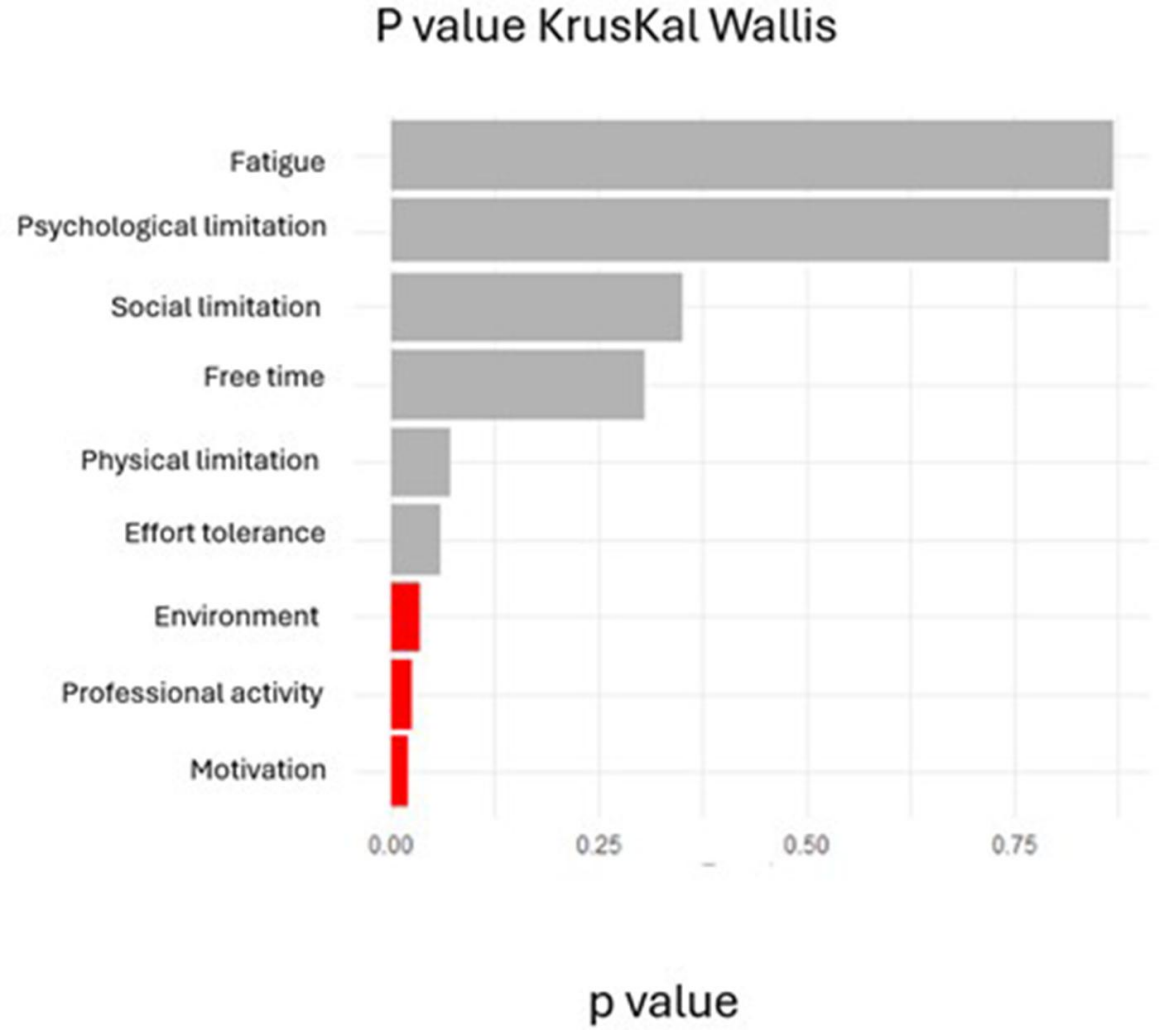
Second round Kruskal-Wallis results comparing barrier scores across clusters (*n* = 13).

Figure 4 displays Kruskal–Wallis test results comparing barrier scores across the three clusters. Significant between-cluster differences (*p* < 0.05) were observed for environmental barriers (H = 7.82, *p* = 0.02), motivation (H = 8.14, *p* = 0.017), and professional obligations (H = 6.93, *p* = 0.031). Exercise tolerance showed a trend toward significance (*p* = 0.056). *post-hoc* Dunn tests (with multiple-comparison adjustment) indicated that Cluster 2 differed significantly from Clusters 1 and 3 on environmental barriers (*p* < 0.05).

Regarding PA behavior, Cluster 2 reported intermediate PA volumes relative to the other groups higher than Cluster 1, in which 50% of participants were completely inactive, yet lower than Cluster 3, whose participants typically engaged in 30-minute sessions on 1–4 days per week. In terms of activity type, Cluster 2 most frequently reported an unstructured PA (occasional independent exercise session), indicating less regular and shorter structured bouts than Cluster 3 and a clearly greater engagement than the inactivity profile characterizing Cluster 1.

## Discussion

This study employs an innovative methodological approach and is among the first to recognize CAD patients as experts in their rehabilitation, filling a key gap in patient-centered CR research. A moderate but meaningful consensus emerged among the expert population CAD patients highlighting principal barriers to PA during their lifelong maintenance Phase. Indeed, the identification of three distinct clusters of CAD patients highlights the presence of clearly differentiated patient profiles, each characterized by distinct barriers.

### Methodological discussion

This study reported a high attrition rate; however, the observed 50% retention (from 26 to 13 participants) is in line with attrition levels commonly reported in Delphi studies. As noted by Atkins et al., attrition rates in classical Delphi designs can vary widely, ranging from 0% to 92%, potentially impacting the validity of consensus outcomes (30). Prior research has demonstrated that online questionnaires tend to yield lower response rates compared to postal surveys (31), highlighting the influence of delivery mode on participant engagement. In addition, several methodological factors such as the sequencing of questions, the time interval between rounds, the duration of each round, the structure and clarity of feedback, and the complexity of reminder procedures have been previously identified as determinants of response rates (32, 33).

As a pilot study, no formal sample size calculation was conducted. Instead, the sample size was determined based on feasibility constraints and the recruitment capacity of the Eastern Pyrenees cardiac rehabilitation network. Most Delphi studies typically involve panels ranging from 8 to 20 participants, with methodological guidelines suggesting that 8 to 23 participants are generally adequate for initial consensus building (20). Ultimately, the panel size should balance methodological rigour with practical constraints such as time, funding, and expert availability.

The decision to conclude the Delphi method effectively achieved consensus after round 2 was methodologically justified by four criteria. First, the achieved coefficient (W = 0.64, *p* < 0.001) exceeded the “moderate-to-substantial” threshold (≥0.50) identified as acceptable for expert-elicitation studies, approaching the “strong” consensus level of 0.70 (21). Second, contemporary guidance indicates two to three rounds typically suffice to balance consensus building with respondent burden (20, 28, 34). Third, the 50% retention rate (13/26) between rounds further supported termination to preserve data quality and prevent participant attrition. Finally, extending the survey risked “forced consensus,” a recognized threat to Delphi validity that methodological reviews urge investigators to avoid (19).

While cluster analysis typically benefits from larger samples, exploratory K-means clustering can be informative even with smaller datasets (*n* ≥ 10) when used cautiously for hypothesis generation rather than definitive classification (35). Given our pilot study design, we employed K-means as an exploratory tool to identify potential patient profiles, with silhouette analysis (coefficient = 0.42) confirming reasonable cluster separation.

### Results discussion

Our predominantly male sample aligns with established French epidemiological patterns indicating a CR rate significantly lower for women than that for men, respectively 14.8% vs. 25.8% (36). The temporal distribution of our sample 53.84% completing rehabilitation within six months and 46.15% between six and twelve months prior is particularly significant given the well-documented evidence of benefit decay following supervised CR. Cardiorespiratory improvements achieved during structured programs have been shown to decline within three months post-completion, with functional capacity potentially returning to baseline within 12 to 18 months in the absence of sustained exercise engagement (37, 38). These findings underscore the critical importance of developing effective strategies to support long-term PA adherence during the transition to lifelong maintenance Phase.

None of the participants in our study engaged in a structured or supervised long-term maintenance phase CR, nor did they report exercising under the supervision of an adapted physical activity professional. This highlights the insufficient implementation of PA prescriptions for CAD patients. In France, available options are indeed limited and mainly rely on community-based initiatives (e.g., Heart and Health club) or the emerging “Sport and Health House” network. Recent initiatives aim to address these gaps, but they remain at an experimental stage (39, 40). In contrast, the United Kingdom offers a more formalized long-term maintenance phase model, with certified instructors ensuring continuity after clinical rehabilitation (41). Strengthening structured physical activity pathways, integrating community programs, and exploring hybrid or digital approaches are essential to improving access to supervised exercise in France.

Initial barrier identification yielded 53 distinct impediments, subsequently consolidated into nine overarching categories. Physical limitations (24.5%), time constraints (13.2%), and motivational challenges (13.2%) emerged as the most frequently cited obstacles. However, round 2 prioritization, achieving moderate yet statistically significant consensus, revealed a different hierarchy: environmental factors, motivation, and exercise tolerance assumed greater importance than initially indicated by frequency alone. While our findings align with previously documented barriers including environmental, physical, motivational, and temporal constraints (9, 41–44), the notable absence of economic barriers distinguishes our cohort. While the Eastern Pyrenees are socio-economically disadvantaged, this divergence probably reflects the fairly comprehensive medical coverage offered by the French system, combined with therapeutic education emphasising low-cost PA alternatives offered during rehabilitation (45, 46).

Motivational barriers emerged as particularly significant, highlighting complex psychological mechanisms beyond simple willingness to engage. According to self-determination theory (48–50), motivation exists along a continuum from extrinsic (driven by external pressures or rewards) to intrinsic (arising from inherent satisfaction). Autonomous motivation, particularly intrinsic forms, demonstrates superior efficacy in sustaining long-term behavioral change (49). Among cardiac populations, key motivational facilitators include perceived competence, health-related goals, enjoyment, and social support (51–53). Notably, participants reporting physical limitations unrelated to CAD may experience reinforced avoidance behavior driven by injury fears, further compromising motivational resources necessary for autonomous engagement. Consequently, interventions fostering intrinsic motivation through autonomy support, competence enhancement, and meaningful rationale provision appear essential for promoting PA adherence in the lifelong maintenance Phase CR.

Environmental barriers unexpectedly emerged as the primary impediment, particularly surprising given southern France's favorable climate and abundant natural spaces. Previous research has documented environmental constraints including neighborhood safety concerns, limited green space access, and adverse weather as particularly relevant for older or vulnerable populations (53). Among participants over 60, environmental barriers correlated with security concerns regarding outdoor PA (54). This prominence of environmental obstacles in an ostensibly conducive setting suggests that perceived barriers may reflect complex psychosocial factors beyond objective environmental characteristics.

Our results revealed complex barrier interactions, suggesting that PA engagement is impeded not by isolated factors but through synergistic barrier networks (55). Cluster 1 participants, primarily limited by exercise intolerance, also reported significant environmental barriers, indicating cumulative effects between physical deconditioning and external constraints. The strong negative correlation between environmental barriers and exercise tolerance (r = −0.68, *p* = 0.01) supports this interpretation: declining physical capacity amplifies environmental obstacle perception. This aligns with the deconditioning spiral concept, whereby sedentary behavior and reduced functional capacity perpetuate effort avoidance (56, 57). The positive correlation between motivation and environmental barriers (r = 0.61) indicates that individuals reporting higher environmental barriers also tended to report higher motivational barriers. It supports a barrier-accumulation pattern whereby environmental constraints and motivational impediments tend to co-occur, potentially amplifying their impact on PA uptake (58). Such clustering of perceived obstacles aligns with our interpretation of complex, mutually reinforcing barrier networks. Cluster 2 exhibited a profile dominated by environmental and motivational barriers, both scoring highly (median = 9), alongside substantial social limitations. Unlike Cluster 1's physical focus, this pattern emphasizes contextual and relational factors, aligning with concepts of motivational vulnerability in unsupportive environments where external obstacles and limited social reinforcement compromise self-regulation and autonomy (53, 54). Cluster 3 revealed organizational and psychological pressures rather than physical or environmental constraints. The convergence of professional demands, time scarcity, and psychological strain suggests “role overload” (59), wherein multiple simultaneous responsibilities exceed available resources. PA becomes deprioritized rather than consciously avoided, as individuals lack requisite mental and emotional capacity. This profile exemplifies how role strain and temporal pressure constitute significant behavioral barriers independent of structural or physiological limitations (60, 61).

### Practical applications

Clustering analysis successfully structured this diversity into meaningful patient profiles. This exploratory technique revealed underlying patterns that basic descriptive statistics would overlook, proving particularly valuable for tailoring interventions to specific barrier constellations (62).

The implementation of this protocol in clinical practice is supported by the typical organizational structure CR programs. In most cases, patients participate in several weeks of supervised phase II CR before transitioning to a lifelong maintenance phase. This care trajectory provides an opportunity to administer a brief, Delphi-based barrier questionnaire at the beginning or end of Phase II. Although the pilot analysis was conducted on a small sample, this approach remains feasible in real-world settings by incorporating the questionnaire into routine clinical procedures. It would allow for the early identification of barriers and the development of individualized follow-up strategies as patients return to their home environment.

The identified clusters demonstrated distinct barrier patterns with important implications for intervention design. Cluster 1 patients, characterized by physical limitations, reduced exercise tolerance, and fatigue. For this group, interventions should support a gradual and reassuring progression, with simple and achievable goals to improve both PA and self-confidence. Enhancing self-efficacy (63) the belief in one's ability to succeed is key, as it strongly influences program adherence. In practice, this may include an initial physiotherapy assessment to ensure safety, progressive home-based programs with video guidance, gradual increases in intensity starting at around 40% of maximum heart rate, and weekly telephone follow-ups. In line with CR guidelines, recommended practice involves 3–5 sessions per week of moderate-intensity PA, beginning with accumulated bouts of 10 min and progressing toward 30–45 min of continuous walking or cycling. Complementary resistance training with light loads and functional exercises can further enhance tolerance and counteract fatigue.

Cluster 2 patients face a mix of environmental barriers, low motivation, and limited social support. Addressing issues such as lack of access to safe places to exercise, transportation challenges, or poor weather is essential to support their participation. Reducing these obstacles can help boost motivation. For this group, it is also important to offer activities that feel both achievable and enjoyable, in line with the self-determination theory (47), which emphasizes the role of intrinsic motivation in sustaining behavior. Research shows that intrinsic motivation is closely linked to long-term adherence (64). In addition, how safe and accessible the environment feels is a key factor influencing PA (65), highlighting the importance of assessing each patient's context to offer tailored solutions. In practice, recommended activity includes accessible group sessions (e.g., walking groups or supervised classes in community centers), ideally 2–3 times per week at moderate intensity, complemented by home-based sessions. Patients should accumulate at least 150 min of moderate-intensity PA per week, which may be organized as 30 min on five days per week or three longer sessions of 60 min.

Patients in cluster 3 are mainly limited by lack of time and organizational challenges. Therefore, it is important to offer flexible and time-efficient exercises. For example, three 10-minute brisk walking sessions per day provide similar cardiovascular and metabolic benefits as one continuous 30-minute session (66). These methods allow patients to accumulate activity without needing long uninterrupted periods. Using coaching apps, integrating activity at work, and involving family members can also make it easier to stay active. Additionally, high-intensity interval training (HIIT) is commonly used in CR; it is safe and improves heart capacity more than moderate continuous exercise (68). For this group, HIIT sessions, such as 4 × 4 min at high intensity with active recovery, can be done 1 to 2 times per week. Other sessions can include brisk walking, stair climbing, or short home circuits. These approaches meet guidelines while fitting into patients' busy schedules.

### Limitations

Several limitations warrant consideration: geographic restriction to Eastern Pyrenees representing a small southern French department, constrains external validity. As a pilot investigation, expansion to multiple Occitanie departments would enhance representativeness, given the region's 120,000 + registered CAD patients. However, Occitanie's 2021 CR admission rate of 17.7% remains suboptimal (36), potentially limiting recruitment; the predominantly male sample (92.31%) inadequately represents gender-specific barriers and preferences documented in the literature (68–70); additionally, the absence of clinical severity indicators prevents analysis of barrier variations across disease stages. Furthermore, the small sample size (*n* = 26 reducing to *n* = 13) limits generalizability positioning our findings as hypothesis-generating rather than confirmatory and requiring validation in larger cohort. Future studies should target *n* ≥ 30 for round 2 to ensure robust clustering. In addition, while the Delphi method effectively achieved consensus, its limitations in exploring deeper motivational underpinnings became apparent (29). The moderate consensus level (W = 0.64), while exceeding established thresholds for operational consensus in health sciences research, highlights considerable heterogeneity in patient experiences during lifelong maintenance Phase CR, necessitating more nuanced analytical approaches.

## Conclusion

Despite its limited scope, this pilot study provides findings that may be of interest to a broader audience beyond the research team, particularly due to the methodological approach used and the practical insights gained regarding protocol implementation. Indeed, the combination of Delphi consensus building and K-means clustering revealed not only prominent impediments but also their organization into distinct patient profiles. These findings underscore the limitations of universal recommendations and advocate for individualized intervention strategies that reflect the multidimensional nature of patient experiences. Taken together, the results provide evidence in support of a fundamental reorganization of lifelong maintenance Phase of cardiac rehabilitation delivery shifting toward personalized, flexible, and context-sensitive follow-up protocols. Sustaining long-term physical activity adherence requires a nuanced understanding of both perceived and actual barriers, in order to effectively support patient engagement beyond the confines of structured rehabilitation programs.

## Data Availability

The raw data supporting the conclusions of this article will be made available by the authors, without undue reservation.
